# Theta functions, broken lines and 2-marked log Gromov-Witten invariants

**DOI:** 10.1007/s00229-025-01640-z

**Published:** 2025-06-19

**Authors:** Tim Gräfnitz

**Affiliations:** Leibniz University Hannover, Institute of Algebraic Geometry, Welfengarten 1, 30167 Hannover, Germany

**Keywords:** 14J33, 14N10, 14T90, 14A21, 14N35, 14K25, 05E40

## Abstract

Theta functions were defined for varieties with effective anticanonical divisor [11] and are related to certain punctured Gromov-Witten invariants [2]. We show that in the case of a log Calabi-Yau surface (*X*, *D*) with smooth very ample anticanonical divisor we can relate theta functions and their multiplicative structure to certain 2-marked log Gromov-Witten invariants. This is a natural extension of the correspondence between wall functions and 1-marked log Gromov-Witten invariants [8]. It gives an enumerative interpretation for the intrinsic mirror construction of [17] and will be related to the open mirror map of outer Aganagic-Vafa branes in [9].

## Introduction

Classically theta functions were defined in the theory of elliptic curves. Mumford generalized their definition to Abelian varieties and studied degenerations of such varieties [22]. In [11] a generalized version of theta functions was defined on varieties with effective anticanonical class. They were used in [10] to construct the mirror to certain maximally degenerate log Calabi-Yau pairs and naturally fit within the Gross-Siebert program of mirror symmetry [13–15]. It was shown in [18], Theorem 4.5, that these theta functions are linked to certain *punctured Gromov-Witten invariants* [2]. This leads to an *intrinsic mirror symmetry* construction [17], where the mirror to a Calabi-Yau variety or log Calabi-Yau pair is constructed from its theta functions or, equivalently, its punctured invariants.

We consider the case complementary to [10] of a log Calabi-Yau pair (*X*, *D*) with *X* a smooth projective surface and *D* a *smooth* very ample anticanonical divisor. We show that in this case we can circumvent the notion of punctured invariants and relate theta functions to 2-marked *log Gromov-Witten invariants*.

### Definition

For an effective curve class $\beta$ on *X* and $p,q\in \mathbb {Z}_{>0}$ with $p+q=D\cdot \beta$ let $\mathscr {M}_{0,1}(X,\beta )_{(p,q)}$ be the moduli space of basic stable log maps to *X* of genus *g*, class $\beta$ and two marked points with contact orders *p* and *q* with *D*. By [16] this a proper Deligne-Mumford stack of virtual dimension 1 and admits a virtual fundamental class $\llbracket \mathscr {M}_{0,1}(X,\beta )_{(p,q)}\rrbracket$. Let $ev : \mathscr {M}_{0,1}(X,\beta )_{(p,q)} \rightarrow D$ be the evaluation map at the marked point of order *p*. Define the 2-marked log Gromov-Witten invariant

$$\begin{aligned} N_{p,q}(X,\beta ) := \int _{\llbracket \mathscr {M}_{0,1}(X,\beta )_{(p,q)}\rrbracket } ev ^\star [pt ] \in \mathbb {Q} \end{aligned}$$

and write

$$\begin{aligned} N_{p,q}(X) := \sum _\beta N_{p,q}(X,\beta ), \end{aligned}$$

where the sum is over all effective curve classes of *X*. We will omit *X* in the notation whenever it is clear from the context.

Consider a *toric degeneration* of *X*, i.e., a degeneration into several toric varieties glued along toric divisors such that the family is strictly semistable away from a codimension 2 locus. If *X* is toric, such a degeneration can be obtained from the central subdivision of the moment polytope by the Mumford construction [22]. The *dual intersection complex* $(B,\mathscr {P},\varphi )$ was defined in [13], §4.2, and consists of an affine manifold with singularities *B*, a polyhedral decomposition $\mathscr {P}$ and a multi-valued piecewise linear function $\varphi$. Since *D* is smooth, the affine manifold *B* has a unique unbounded direction $m_{\text {out}}\in \Lambda _{B_0}$ (corresponding to *D*), where $B_0=B\setminus \text {Sing}(B)$ is the complement of the affine singular locus and $\Lambda _{B_0}$ is the sheaf of integral tangent vectors. By the constructions in [15] and [11], the dual intersection complex induces a wall structure (or scattering diagram) $\mathscr {S}$ and broken lines – piecewise linear maps $\mathfrak {b} : [0,\infty ) \rightarrow B$ with a monomial $a_iz^{m_i}$ attached to every linear segment such that $m_i=(\bar{m}_i,h_i)$ with $\bar{m}_i$ parallel to the segment and $a_iz^{m_i}$, $a_{i+1}z^{m_{i+1}}$ are related by wall crossing in $\mathscr {S}$.

For $q\in \mathbb {N}$ and a point $P\in B_0=B\setminus \text {Sing}(B)$, there is a theta function defined by

$$\begin{aligned} \vartheta _q(P) = \sum _{\mathfrak {b}\in \mathfrak {B}_q(P)} a_{\mathfrak {b}} z^{m_{\mathfrak {b}}}, \end{aligned}$$

where $\mathfrak {B}_q(P)$ is the set of broken lines for $\mathscr {S}$ with endpoint $\mathfrak {b}(0)=P$ and monomial attached to the unbounded line segment given by $z^{(qm_{\text {out}},q)}$, and $a_{\mathfrak {b}}z^{m_{\mathfrak {b}}}$ is the monomial attached to the line segment ending in *P*.

By [11], Theorem 3.24, theta functions generate a ring with multiplication rule

$$\begin{aligned} \vartheta _p(P) \cdot \vartheta _q(P) = \sum _{r=0}^\infty \alpha _{p,q}^r(P) \vartheta _r(P) \end{aligned}$$

with structure constants

$$\begin{aligned} \alpha _{p,q}^r(P) = \sum _{\begin{array}{c} (\mathfrak {b}_1,\mathfrak {b}_2)\in \mathfrak {B}_p(P)\times \mathfrak {B}_q(P) \\ m_{\mathfrak {b}_1}+m_{\mathfrak {b}_2}= \ rm_{\text {out}} \end{array}} a_{\mathfrak {b}_1}a_{\mathfrak {b}_2}. \end{aligned}$$

The polyhedral decomposition $\mathscr {P}$ of *B* is a set of (possibly unbounded) polytopes called *cells*. It contains one bounded maximal cell and several unbounded maximal cells. Let *P* be a point infinitely far away from the bounded maximal cell. Let $m_{out }$ be the unbounded direction and write $x=z^{(-m_{out },-1)}$ and $t=z^{(0,1)}$. Note that at *P* we have $\varphi (-m_{out })=-1$, so *x* has *t*-order zero.

### [Style2 Style2 Style2]Theorem 1

We have

$$\begin{aligned} \vartheta _q(P) = x^{-q} + \sum _{p=1}^\infty pN_{p,q}t^{p+q}x^p. \end{aligned}$$

The reason for this equation is as follows. As we move *P* away from the bounded maximal cell of *B* the slope^1^ of the walls in $\mathscr {S}$ increases. If we move sufficiently far away all broken lines ending in *P* have to be parallel to the unbounded direction (Proposition 2.5). We can complete such a broken line to a tropical curve with two unbounded legs. One of these legs contains *P*, corresponding to a fixed point. Then the tropical correspondence theorem for log Calabi-Yau pairs with smooth divisor [8] (more precisely, an extension of it incluing point conditions) gives a relation between broken lines and 2-marked log Gromov-Witten invariants, leading to the above correspondence for theta functions. By a similar reason we have the following.

### [Style2 Style2 Style2]Theorem 2

The multiplication rule for theta functions is given by

$$\begin{aligned} \vartheta _p \cdot \vartheta _q = \vartheta _{p+q} + \sum _{r=0}^{\text {max}\{p,q\}-1} \alpha _{p,q}^r \vartheta _r \end{aligned}$$

with structure constants (we define $N_{p,q}(X)=0$ when $p \le 0$ or $q\le 0$)

$$\begin{aligned} \alpha _{p,q}^r = \left( (p-r)N_{p-r,q} + (q-r)N_{q-r,p}\right) t^{p+q-r}. \end{aligned}$$

Comparing this with Theorem 1 we obtain relations between the numbers $N_{p,q}$. These determine all $N_{p,q}$ from the invariants $N_{1,q}$ with fixed point multiplicity 1.

### Remark

By [9], Corollary 6.6, the invariants $N_{1,q}(X,\beta )$ agree with 1-marked invariants $N_q(\hat{X},\pi ^\star \beta -C)$ of the blow up at a point $\pi : \hat{X} \rightarrow X$, where *C* is the exceptional divisor. Using this relation, Theorem 2 above, the log-local correspondence of [7] and results of [20] we show in [9] that $\vartheta _1$ agrees with the open mirror map of framing-0 outer Aganagic-Vafa branes on the local Calabi-Yau threefold given by the total space of the canonical bundle $K_X$.

### Remark

In [17] the structure constants were defined as

$$\begin{aligned} \alpha _{p,q}^r = \sum _\beta N_{pq}^r(\beta )t^\beta \end{aligned}$$

where the sum is over all effective curve classes $\beta$ of *X* and $N_{pq}^r(\beta )$ are certain punctured Gromov-Witten invariants of class $\beta$. In particular $\alpha _{m_1,m_2}^m$ does not depend on *P*. It was shown in [18], Theorem 4.5, that this agrees with the above definition of $\alpha _{p,q}^r(P)$. In combination with Theorem 2 we obtain a triangle of relations

### Glossary

- (*X*, *D*) a log Calabi-Yau pair
  - *X* a smooth projective surface with very ample anticanonical bundle
  - *D* a smooth anticanonical divisor of *X*
- $N_{p,q}(X,\beta )$ the logarithmic Gromov-Witten invariant of *X* of genus 0 and class $\beta$ with 2 marked points with a prescribed point of contact order *p* and an unprescribed point of contact order *q* with *D*.

§1

- $(\check{B},\check{\mathscr {P}},\check{\varphi })$ the intersection complex of a toric degeneration $\mathfrak {X}$ of (*X*, *D*)
- $(B,\mathscr {P},\varphi )$ the dual intersection complex of $\mathfrak {X}$ ([13], §4)
  - *B* an integral affine manifold with singularities ([13], §1)
  - $\mathscr {P}$ a polyhedral decomposition of *B* ([13], §1)
  - $\varphi :B\rightarrow \mathbb {Z}$ a multi-valued piecewise linear function, strictly convex with respect to $\mathscr {P}$ ([13], §1)
- $m_{\text {out}}$ the unique unbounded direction of *B*
- $\mathscr {S}_k$ the wall structure (scattering diagram) consistent order *k* ([15], §3)
  - walls $\mathfrak {p}$ with primitive integral tangent vector $m_{\mathfrak {p}}$, normal vector $n_{\mathfrak {p}}$

§2

- $\mathfrak {B}_{q}(P)$ the set of broken lines $\mathfrak {b}:[0,\infty )\rightarrow B$ for $\mathscr {S}_\infty$ with endpoint $\mathfrak {b}(0)=P$ and incoming monomial $z^{(qm_{\text {out}},q)}$ (Definition 2.1; [11], §3)
  - $\mathfrak {B}^{(k)}_{q}(P)\subset \mathfrak {B}_{q}(P)$ the subset of broken lines of order $\le k$
  - $\mathfrak {B}^\circ _{p,q}(P)\subset \mathfrak {B}^{(p+q)}_{q}(P)$ the subset of broken lines with ending monomial $a_{\mathfrak {b}}z^{(-pm_{\text {out}},q)}$, with $a_{\mathfrak {b}}\in \mathbb {N}$

§3

- $\mathfrak {H}^\circ _{q}(P)$ the set of tropical disks $h^\circ :\Gamma \rightarrow B$ with univalent vertex $V_\infty$ mapping to *P* and one unbounded leg of weight *q* (Definition 3.1)
- $\mathfrak {H}^\circ _{p,q}(P)\subset \mathfrak {H}^\circ _{q}(P)$ the subset of tropical disks such that the edge $E_\infty$ adjacent to $V_\infty$ has weight vector $u_{(V_\infty ,E_\infty )}=-pm_{\text {out}}$
- $\mathfrak {H}_{p,q}(P)$ the set of tropical curves $h:\Gamma \rightarrow B$ with two unbounded legs of weights *p*, *q*, the first meets a point *P* in an unbounded chamber of $\mathscr {S}_{p,q}$

§4

- $(\tilde{B},\mathscr {P},\varphi )$ the dual intersection complex of a log resolution $\tilde{\mathfrak {X}}$
- $\tilde{\mathfrak {H}}_{p,q}(P)$ the set of tropical curves $\tilde{h}:\tilde{\Gamma }\rightarrow \tilde{B}$ with two unbounded legs of weights *p*, *q*, the first meets a point *P* in an unbounded chamber of $\mathscr {S}_{p,q}$
- $V_{(I)}(\tilde{\Gamma }),V_{(II)}(\tilde{\Gamma }),V_{(III)}(\tilde{\Gamma })$ set of vertices of $\tilde{\Gamma }$ of given type (Definition 4.5)
- $N_V$ the contribution of a vertex $V\in \tilde{\Gamma }$ to $N_{p,q}(X,\beta )$ (Definition 4.14)
- $N_V^{\text {tor}}$ the toric Gromov-Witten invariant $N_{\textbf{m}}(\textbf{w})$ of [12], Definition 3.1

§5

- $\vartheta _q(P)$ the theta function defined by broken lines in $\mathfrak {B}_{q}(P)$ (Definition 5.1)

## Toric degenerations and wall structures

Tropical geometry is a piecewise linear version of algebraic geometry, hence naturally linked to combinatorics. The most natural class of varieties on which to consider tropical geometry are toric varieties. They admit a combinatorial description in terms of the orbits of their torus action. This can be made explicit in terms of a fan or, dually and given a polarization, a “momentum” polytope. A similar combinatorial description for a non-toric variety *X* is obtained via a toric degeneration $\mathfrak {X}$ – a degeneration whose central fiber $X_0$ is a union of toric varieties glued along toric divisors and such that the family is strictly semistable away from a codimension 2 subset. See [13] for more details.

The *dual intersection complex* $(B,\mathscr {P})$ of $\mathfrak {X}$ is obtained by gluing together the fans of the irreducible components of $X_0$ according to the combinatorics of their intersection. Locally at a vertex such a fan induces an affine structure on *B*. An affine structure on maximal cells is given by the local structure of the family $\mathfrak {X}$ at the corresponding point. This is an affine toric variety defined by a cone over a polytope, and this polytope gives the affine structure on the maximal cell. These affine structures need not fit together, so *B* is an affine manifold with singularities. It is glued from (possibly unbounded) polytopes (called cells) and $\mathscr {P}$ is a collection of these. In the particular case, $\mathscr {P}$ contains exactly one unbounded maximal cells. A polarization of *X* corresponds to a strictly convex multi-valued piecewise linear function ([13], Definition 1.47) $\varphi$ on $(B,\mathscr {P})$. The anticanonical polarization corresponds to $\varphi$ defined by the conditions that it is zero on the bounded maximal cells, has slope 1 along the unbounded edges of $\mathscr {P}$ and its domains of affine linearity are given by the maximal cells of $\mathscr {P}$.

The *intersection complex* $(\check{B},\check{\mathscr {P}},\check{\varphi })$ is obtained by gluing together momentum polytopes of the irreducible components of $X_0$. To do so, we need a polarization on *X*. In this case the multi-valued piecewise linear function $\check{\varphi }$ describes the family $\mathfrak {X}$ locally: its momentum polytope is the upper convex hull of $\check{\varphi }$.

### [Style3 Style3]Example 1.1

A toric degeneration of $(\mathbb {P}^2,E)$, for *E* an elliptic curve, is given by

$$\begin{aligned} \mathfrak {X}= &   \{XYZ=t^3(W+f_3)\} \subset \mathbb {P}(1,1,1,3)\times \mathbb {A}^1 \rightarrow \mathbb {A}^1, \\ \mathfrak {D}= &   \{W=0\} \subset \mathfrak {X}. \end{aligned}$$

**Fig. 1:**
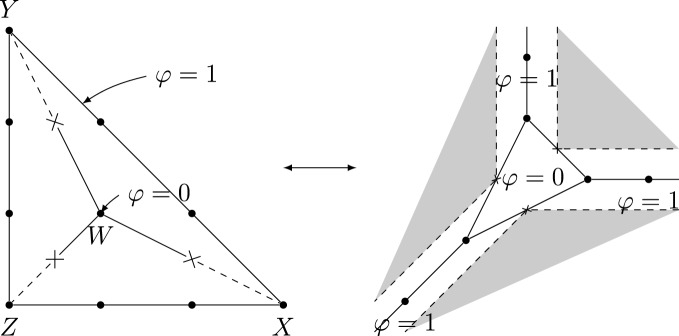
The intersection complex (left) of $(\mathbb {P}^2,E)$ and its dual (right)

Here *W* is the variable in $\mathbb {P}(1,1,1,3)$ of weight 3, *t* is the variable of $\mathbb {A}^1$ and the map is given by projection to *t*. Moreover, $f_3$ is a homogeneous degree 3 polynomial in *X*, *Y*, *Z*, general in the sense that $XYZ/t^3-f_3$ is nonsingular for general *t*. Indeed, for $t\ne 0$ we can solve for $W=XYZ/t^3-f_3$, so the general fiber of $\mathfrak {X}$ is $X=\mathbb {P}^2$ and the general fiber of $\mathfrak {D}$ is an elliptic curve *E* by the generality condition on $f_3$. For $t=0$ we have $XYZ=0$, so $X_0$ is a union of three $\mathbb {P}(1,1,3)$ glued along toric divisors. Figure 1 shows the intersection complex and its dual for this toric degeneration.

It is natural to ask whether one can go back and construct a toric degeneration $\check{\mathfrak {X}}$ from a triple $(B,\mathscr {P},\varphi )$ as above such that $(B,\mathscr {P},\varphi )$ is the intersection complex of $\check{\mathfrak {X}}$. This was solved under some assumptions in [15] and involves the iterative construction of wall structure via scattering calculations. A *wall* is a codimension 1 polyhedral subset $\mathfrak {p}$ of *B* with some attached function $f_{\mathfrak {p}}$ living in the ring $R_\varphi$ defined by

$$\begin{aligned} P_\varphi= &   \{m=(\bar{m},h)\in M\oplus \mathbb {Z} \ | \ h \ge \varphi (\bar{m})\}, \\ R_\varphi= &   \varprojlim \mathbb {C}[P_\varphi ]/(t^k), \quad t=z^{(0,1)}. \end{aligned}$$

A wall defines an automorphism of some localization of $R_\varphi$ by

$$\begin{aligned} \theta _{\mathfrak {p}}(z^{(\bar{m},h)}) = f_{\mathfrak {p}}^{-{\left\langle {n_{\mathfrak {p}},\bar{m}}\right\rangle }}z^{(\bar{m},h)}. \end{aligned}$$

Here $n_{\mathfrak {p}}$ is a primitive normal vector to $\mathfrak {p}$ (this involves a choice of orientation). We restrict to the 2-dimensional case such that walls are 1-dimensional.

The initial wall structure $\mathscr {S}_0$ consists of walls coming out of the affine singularities of *B* with attached functions $1+z^{(\bar{m},0)}$, where $\bar{m}$ is the direction of $\mathfrak {p}$. Scattering means whenever two or more walls intersect they produce more walls with base at the intersection, such that the clockwise composition of the automorphisms $\theta _{\mathfrak {p}}$ is the identity. For any finite *t*-order *k* this produces finitely many new rays, leading to a wall structure $\mathscr {S}_k$ that is “consistent to order *k*”. The formal limit for $k\rightarrow \infty$ is denoted by $\mathscr {S}_\infty$. Note that the *t*-order of $z^{(\bar{m},h)}$ at a point $x\in B$ is $\varphi _x(-\bar{m})+h\ge 0$, where $\varphi _x$ is the linear function that equals $\varphi$ on the cell of $\mathscr {P}$ containing *x*, since $z^{(\bar{m},h)} = \left( z^{(-\bar{m},\varphi _x(-\bar{m}))}\right) ^{-1} t^{\varphi _x(-\bar{m})+h}.$

### [Style3 Style3]Example 1.2

**Fig. 2:**
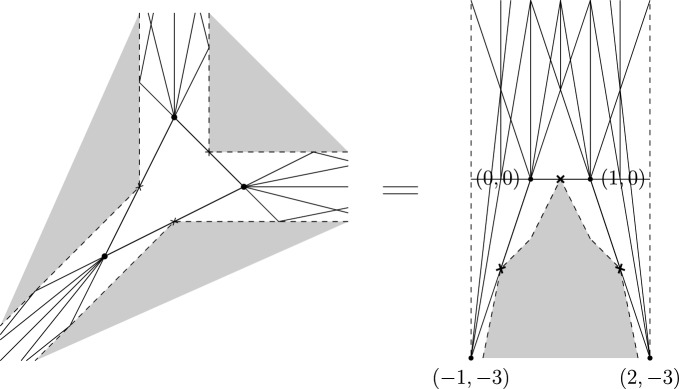
The wall structure for $(\mathbb {P}^2,E)$ of order $2\cdot 3=6$

Figure 2 shows the wall structure consistent to *t*-order $2\cdot 3=6$ for $(\mathbb {P}^2,E)$ in two different charts of the affine structure. There are functions attached to each of these walls, but we don’t show them for readability.

The idea of the Gross-Siebert program is that the family $\check{\mathfrak {X}}$ constructed this way is a degeneration of the mirror to (*X*, *D*). As is well known, the mirror to a log Calabi-Yau pair (*X*, *D*) should be a Landau-Ginzburg model, that is, a toric variety *Y* together with a ”superpotential” $W : Y \rightarrow \mathbb {C}$ whose critical locus is compact. It is shown in [6] that such a superpotential can be constructed from broken lines, see §2. For more details on these constructions see [13–15].

## Broken lines

Let *X* be a smooth projective surface with very ample anticanonical bundle and let *D* be a smooth anticanonical divisor. As in §1, let $(B,\mathscr {P},\varphi )$ be the dual intersection complex of a toric degeneration of the log Calabi-Yau pair (*X*, *D*).

### [Style1 Style1]Definition 2.1

A *broken line* for a wall structure $\mathscr {S}$ on $(B,\mathscr {P})$ is a proper continuous map

$$\begin{aligned} \mathfrak {b} : (-\infty ,0] \rightarrow B_0 \end{aligned}$$

with image disjoint from any joints of $\mathscr {S}$, along with a sequence $-\infty =t_0<t_1<\cdots <t_r=0$ for some $r\ge 1$ with $\mathfrak {b}(t_i)\in |\mathscr {S}|$ for $i\le r-1$, and for each $i=1,\ldots ,r$ an expression $a_iz^{m_i}$ with $a_i\in \mathbb {C}\setminus \{0\}$, $\bar{m}_i\in \Lambda _{\mathfrak {b}(t)}$ for any $t\in (t_{i-1},t_i)$, defined at all points of $\mathfrak {b}([t_{i-1},t_i])$, and subject to the following conditions:

1. $\mathfrak {b}|_{(t_{i-1},t_i)}$ is a non-constant affine map with image disjoint from $|\mathscr {S}|$, hence contained in the interior of a unique chamber $\mathfrak {u}_i$ of $\mathscr {S}$, and $\mathfrak {b}'(t)=-\bar{m}_i$ for all $t\in (t_{i-1},t_i)$.
2. For each $i=1,\ldots ,r-1$ the expression $a_{i+1}z^{m_{i+1}}$ is a result of transport of $a_iz^{m_i}$ from $\mathfrak {u}_i$ to $\mathfrak {u}_{i+1}$, i.e., is a summand in the expansion of $\theta _{\mathfrak {u}_i\mathfrak {u}_{i+1}}(a_iz^{m_i})$.
3. $a_1=1$ and $m_1=(\bar{m}_1,h)$ has *t*-order zero at $\mathfrak {b}(t_1)$, i.e., $h=\varphi (\bar{m}_1)$.

Write $a_{\mathfrak {b}}z^{m_{\mathfrak {b}}}$ for the ending monomial $a_rz^{m_r}$.

Since *D* is smooth, *B* has a unique unbounded direction $m_{out }$. Since *D* is anticanonical, $\varphi$ is zero on the bounded maximal cell and $\varphi (m_{out })=1$ on all unbounded cells. Let $\mathscr {S}_\infty$ be the consistent wall structure defined by $(B,\mathscr {P},\varphi )$. Then $\varphi (m_{out })=1$ along all walls of $\mathscr {S}_\infty$, since walls are not contained in the interior of the bounded maximal cell ([8], Lemma 5.14). Hence, each broken line for $\mathscr {S}_\infty$ has asymptotic monomial $m_1=(qm_{out },q)$ for some $q\in \mathbb {N}$.

### [Style1 Style1]Definition 2.2

For a point $P\in B_0$ let $\mathfrak {B}(P)$ be the set of be the set of broken lines for the consistent wall structure $\mathscr {S}_\infty$ with endpoint $\mathfrak {b}(0)=P$.

For $P\in B_0$ and $q\in \mathbb {N}$ let $\mathfrak {B}_q(P)$ be the set of broken lines in $\mathfrak {B}(P)$ with asymptotic monomial $m_1=(qm_{out },q)$.

Write $\mathfrak {B}_q^{(k)}(P)$ for the subset of $\mathfrak {B}_q(P)$ consisting of walls such that the ending monomial $a_{\mathfrak {b}}z^{m_{\mathfrak {b}}}$ has *t*-order $\le k$. This means if $m_{\mathfrak {b}}=(\bar{m},h)$, then $\varphi (-\bar{m}) \le k-h$.

Note that broken lines in $\mathfrak {B}^{(k)}_q(P)$ only break at walls of $\mathscr {S}_k$.

### [Style1 Style1]Definition 2.3

For a point $P\in B_0=B\setminus Sing (B)$, define the *superpotential to order k at P* by

$$\begin{aligned} W^k(P) = \sum _{\mathfrak {b}\in \mathfrak {B}^{(k)}(P)} a_{\mathfrak {b}}z^{m_{\mathfrak {b}}}. \end{aligned}$$

### [Style2 Style2]Proposition 2.4

([6], Lemma 4.7) For a given order *k* and chamber $\mathfrak {u}$ of $\mathscr {S}_k$, the superpotential $W^k(P)$ is the same for all general (not contained in a certain lower-dimensional subset) points $P\in \mathfrak {u}$.

### [Style2 Style2]Proposition 2.5

([9], Proposition 3.5) Let $\mathfrak {b}$ be a broken line in $\mathfrak {B}_q^{(k)}(P)$. If *P* lies in an unbounded chamber of $\mathscr {S}_k$, then $\bar{m}_{\mathfrak {b}}$ is parallel to $m_{out }$.

### [Style1 Style1]Definition 2.6

For a point *P* in an unbounded chamber of $\mathscr {S}_{p+q}$, let $\mathfrak {B}_{p,q}(P)$ be the set of broken lines $\mathfrak {b}$ with endpoint $\mathfrak {b}(0)=P$, asymptotic direction $\bar{m}_1=qm_{out }$, and ending direction $\bar{m}_{\mathfrak {b}}=-pm_{out }$.

### Lemma 2.7

For a broken line $\mathfrak {b}$ in $\mathfrak {B}_{p,q}(P)$ we have $m_1=(qm_{out },q)$ and $m_{\mathfrak {b}}=(-pm_{out },q)$. In particular, the ending monomial $a_{\mathfrak {b}}z^{m_{\mathfrak {b}}}$ has *t*-order $p+q$.

### Proof

We have $m_1=(qm_{out },q)$ because $z^{m_1}$ has *t*-order zero by the definition of broken lines. Since all walls in $\mathscr {S}_\infty$ are of the form $1+a_{\mathfrak {p}}z^{(m_{\mathfrak {p}},0)}$, i.e., with zero as second component of the exponent, the ending monomial of $\mathfrak {b}$ is $a_{\mathfrak {b}}z^{m_{\mathfrak {b}}}=a_{\mathfrak {b}}z^{(-pm_{out },q)}$. Hence, the *t*-order of its ending monomial is $\varphi (pm_{out })+q=p+q$. $\square$

### Lemma 2.8

Let *P* be a point in an unbounded chamber of $\mathscr {S}_k$. Then

$$\begin{aligned} \mathfrak {B}_q^{(k)}(P) = \coprod _{p=1}^{k-q} \mathfrak {B}_{p,q}(P). \end{aligned}$$

### Proof

By Proposition 2.5 we have $\mathfrak {B}_q^{(k)}(P) \subset \coprod _{p=1}^{\infty } \mathfrak {B}_{p,q}(P)$. By Lemma 2.7 we have $\mathfrak {B}_{p,q}(P) \subset \mathfrak {B}_q^{(k)}(P)$ if and only if $p+q\le k$. Clearly, for different *p* the sets $\mathfrak {B}_{p,q}(P)$ are disjoint. $\square$

### [Style2 Style2]Proposition 2.9

Let *P* be a point in an unbounded chamber of $\mathscr {S}_{p+q}$. The set $\mathfrak {B}_{p,q}(P)$ is finite for all $p,q\in \mathbb {N}$.

### Proof

By Lemma 2.8 we have $\mathfrak {B}_{p,q}(P) \subset \mathfrak {B}_q^{(p+q)}(P)$. The set $\mathfrak {B}_q^{(k)}(P)$ is finite for all *k*, since all broken lines in $\mathfrak {B}_q^{(k)}(P)$ break at walls of $\mathscr {S}_k$, there are finitely many such walls, and each breaking increases the *t*-order. So $\mathfrak {B}_{p,q}(P)$ is finite. $\square$

## Tropical disks and curves

Roughly speaking, a tropical curve ([8], Definition 3.3) is a piecewise linear embedding $h:\Gamma \rightarrow B$ of a weighted graph $\Gamma$ without 1- or 2-valent vertices but with some 1-vertex edges called legs. The image of a leg must either be unbounded or end in an affine singularity of *B*. Tropical curves fulfill the (ordinary) balancing condition $\sum _{E\ni V} u_{(V,E)}=0$ at vertices *V*, if not specified otherwise. Here $u_{(V,E)}$ is the weight vector of the flag (*V*, *E*), defined in [8], Definition 3.3. We only consider the case when the graph $\Gamma$ is a tree.

### [Style1 Style1]Definition 3.1

A *tropical disk* $h^\circ : \Gamma \rightarrow B$ is a tropical curve with a unique univalent vertex $V_\infty$, such that $h^\circ$ is balanced for all vertices $V \ne V_\infty$.

Write $\mathfrak {H}_q^\circ (P)$ for the set of all rational tropical disks on *B* with a unique unbounded leg, which has weight *q*, and $h^\circ (V_\infty )=P$. Let $\mathfrak {H}_{p,q}^\circ (P)$ be the set of tropical disks in $\mathfrak {H}_q^\circ (P)$ with $u_{(V_\infty ,E_\infty )}=-pm_{out }$, where $E_\infty$ is the unique edge adjacent to $V_\infty$.

### [Style2 Style2]Proposition 3.2

([6], Lemma 6.4) There is a surjective map

$$\begin{aligned} \mu : \mathfrak {H}^\circ _q(P) \rightarrow \mathfrak {B}_q(P) \end{aligned}$$

such that for each $\mathfrak {b}\in \mathfrak {B}_q(P)$ the preimage $\mu ^{-1}(\mathfrak {b})$ is finite. We say a tropical disk in $\mu ^{-1}(\mathfrak {b})$ is obtained from $\mathfrak {b}$ by disk completion.

The map $\mu$ in Proposition 3.2 is defined as follows. Let $h^\circ :\Gamma \rightarrow B$ be a tropical disk in $\mathfrak {H}^\circ _q(P)$. Since $\Gamma$ is a tree, there is a unique path from the vertex $V_\infty$ to the unbounded leg. The edges of this path form the line segments of a broken line $\mathfrak {b}$. Indeed, the breaking happens only at walls of $\mathscr {S}$, since at any vertex of $h^\circ$ there is one adjacent edge whose image is contained in a wall. Conversely, the preimages of $\mathfrak {b}$ are obtained by adding in edges, for each wall $\mathfrak {p}$ at which $\mathfrak {b}$ breaks and for all ancestors of $\mathfrak {p}$, meaning all walls that contribute to the production of $\mathfrak {p}$ in the scattering procedure. With the correct weights, the result will be balanced, i.e., a tropical disk. Instead of adding an edge with weight *w*, we could also add several edges whose sum of weights equals *w*. Hence, there are finitely many preimages of $\mathfrak {b}$, corresponding to partitions of the edge weights.

Let *P* be a general point in an unbounded chamber of $\mathscr {S}_{p+q}$. Since $\mu$ maps tropical disks in $\mathfrak {H}_{p,q}^\circ (P)$ to broken lines in $\mathfrak {B}_{p,q}(P)$, we have the following.

### Corollary 3.3

There is a surjective map with finite preimages

$$\begin{aligned} \mu : \mathfrak {H}_{p,q}^\circ (P) \rightarrow \mathfrak {B}_{p,q}(P). \end{aligned}$$

### Lemma 3.4

We have a decomposition

$$\begin{aligned} \mathfrak {H}_q^\circ (P) = \coprod _{p=1}^\infty \mathfrak {H}_{p,q}^\circ (P). \end{aligned}$$

In particular, for a tropical disk in $\mathfrak {H}_{p,q}^\circ (P)$ the edge $E_\infty$ is parallel to $m_{out }$.

### Proof

The first statement follows from the definitions of $\mathfrak {H}_q^\circ (P)$ and $\mathfrak {H}_{p,q}^\circ (P)$. The second statement follows from Proposition 2.5 and the construction of $\mu$. $\square$

### [Style1 Style1]Definition 3.5

Let $\mathfrak {H}_{p,q}(P)$ be the set of tropical curves on *B* having two unbounded legs, of weight *p* and *q*, and such that the image of the unbounded leg of weight *p* contains *P*.

### [Style2 Style2]Proposition 3.6

There is a bijective map

$$\begin{aligned} \mathfrak {H}^\circ _{p,q}(P) \rightarrow \mathfrak {H}_{p,q}(P), h^\circ \mapsto h \end{aligned}$$

### Proof

By Proposition 2.5 and Corollary 3.3, tropical disks in $\mathfrak {H}^\circ _{p,q}(P)$ have ending edge $E_\infty$ parallel to $m_{out }$. We obtain a tropical curve in $\mathfrak {H}_{p,q}(P)$ by completing $E_\infty$ to an unbounded leg $E_{out }$. $\square$

### Corollary 3.7

There is a surjective map with finite preimages

$$\begin{aligned} \mu : \mathfrak {H}_{p,q}(P) \rightarrow \mathfrak {B}_{p,q}(P). \end{aligned}$$

### Proof

The map is given by composing the inverse of the map from Proposition 3.6 with the map $\mu$ from Proposition 3.2. $\square$

### Corollary 3.8

The set $\mathfrak {H}_{p,q}(P)$ is finite.

**Fig. 3:**
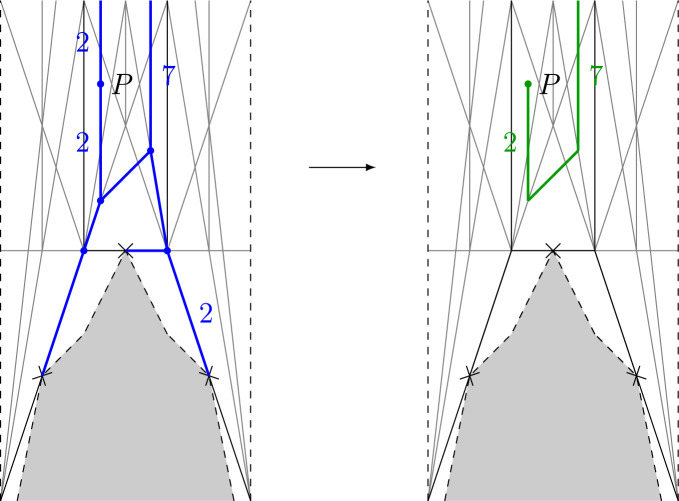
An example of the map $\mu : \mathfrak {H}_{2,7}(P) \rightarrow \mathfrak {B}_{2,7}(P)$. The broken line (green) on the RHS has two preimages. One is the tropical curve (blue) on the LHS. The other is obtained from the tropical curve on the LHS by splitting the bottom right leg with weight 2 into two legs with weight 1 each

### [Style1 Style1]Definition 3.9

Let $h : \Gamma \rightarrow B$ be a tropical curve. Write $V(\Gamma )$ for the set of vertices of $\Gamma$ and $L_\Delta (\Gamma )$ for the set of bounded legs, which necessarily have to end in affine singularities of *B*. For a trivalent vertex $V\in V(\Gamma )$ define, with $u_{(V,E)}\in \iota _\star \Lambda _{h(V)}$ as in [8], Definition 3.3,

$$\begin{aligned} m_V=|u_{(V,E_1)}\wedge u_{(V,E_2)}|=|\text {det}(u_{(V,E_1)}|u_{(V,E_2)})|, \end{aligned}$$

where $E_1,E_2$ are any two edges adjacent to *V*. For a vertex $V\in V(\Gamma )$ of valency $\nu _V>3$ let $h_V$ be the one-vertex tropical curve describing *h* locally at *V* and let $h'_V$ be a deformation of $h_V$ to a trivalent tropical curve. This deformation has $\nu _V-2$ vertices. Define

$$\begin{aligned} m_V=\prod _{V'\in V(h'_V)}m_{V'} \end{aligned}$$

and, by Proposition 2.7 in [12], this expression is independent of the deformation $h'_V$, hence well-defined. For a bounded leg $E\in L_\Delta (\Gamma )$ with weight $w_E$ define

$$\begin{aligned} m_E=\frac{(-1)^{w_E+1}}{w_E^2}. \end{aligned}$$

We define the *multiplicity* of *h* to be

$$\begin{aligned} m_h = \frac{1}{|\text {Aut}(h)|} \cdot \prod _V m_V \cdot \prod _{E\in L_\Delta (\Gamma )} m_E. \end{aligned}$$

The *class* $\beta$ of a tropical curve *h* is the class of the corresponding stable log map. For tropical curves not mapping to the bounded maximal cell $\sigma _0$ it can be read off from the tropical curve via projection to the unbounded direction or, equivalently, via deformation of the toric degeneration, as in [8], §3.4. But tropical curves in $\mathfrak {H}_{p,q}$ may have image intersecting $\sigma _0$, so we need another way to read off $\beta$ from *h*.

In the dual intersection complex *B* of the toric degeneration $\mathfrak {X}\rightarrow \mathbb {A}^1$ there are certain *elementary tropical curves*. They have one unbounded leg with image given by an unbounded edge of *B* and two bounded legs with weight 1. Projection to the unbounded direction or deformation of the toric degeneration as in [8], §3.4, easily shows that they correspond to the toric divisors of $X_0$. Now given any tropical curve $h : \Gamma \rightarrow B$ we can read of the class of *h* from its intersection with the elementary tropical curves. If the intersection is not transversal we can use the moving lemma of tropical intersection theory and consider a small translation of one of the curves. For tropical curves $\tilde{h} : \tilde{\Gamma } \rightarrow \tilde{B}$ the class can be read off from any of its preimages under the map from Lemma 4.6. This is well-defined, since different preimages correspond to different edge splittings without changing the total weight, and this does not affect the intersection multiplicity.

### [Style3 Style3]Remark 3.10

*(Tropical cycles)* More formally, the “elementary tropical curves” should be seen as tropical cocycles in $H^1(\overline{B},\iota _\star \check{\Lambda }_N)$ and the “intersection” is given by an extension of the pairing $H_1(B,\iota _\star \Lambda )\otimes H^1(B,\iota _\star \check{\Lambda })\rightarrow \mathbb {Z}^2$ from [23]. Here $\overline{B}$ is a compactification of *B* and $\check{\Lambda }_N$ is an extension of $\Lambda$ such that the stalk of $\Lambda _N$ at a point in $\overline{B}\setminus B$ is generated by the $m_{\text {out}}$. It follows from [24] there is a natural isomorphism $H^1(\overline{B},\iota _\star \check{\Lambda }_N)\cong \text {Pic}(X)$. This gives the toric divisor (line bundle) corresponding to the “elementary tropical curve”. For a more detailed discussion see §3 of [9].

### [Style1 Style1]Definition 3.11

Let $\mathfrak {H}_{p,q}^\beta (P)$ be the set of tropical curves in $\mathfrak {H}_{p,q}(P)$ of class *P*. Let $\mathfrak {B}_{p,q}^\beta (P)$ be the image of $\mathfrak {H}_{p,q}^\beta (P)$ under $\mu$.

### [Style3 Style3]Example 3.12

**Fig. 4:**
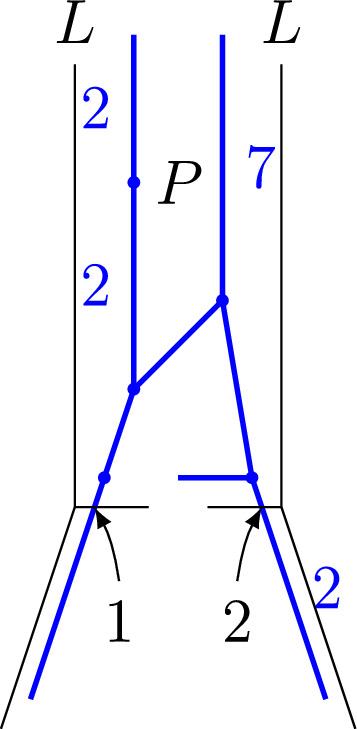
The tropical curve from Fig. 3 has class 3*L*

Consider the tropical curve from Fig. 3. It has nontrivial intersection with two elementary tropical curves, both of them corresponding to a line *L* in $\mathbb {P}^2$. The intersection multiplicities are 1 and 2, respectively, as shown in Fig. 4. Hence, the tropical curve corresponds to curve class $L+2L=3L$ on $\mathbb {P}^2$.

## Tropical correspondence

In [8] the author established a correspondence between wall functions and 1-marked log Gromov-Witten invariants. Here we prove a similar correspondence between broken lines and 2-marked log Gromov-Witten invariants. The steps of the proof are the same, corresponding to the subsections of this section:

1. resolve the log singularities to obtain a log smooth degeneration;
2. refine $\mathscr {P}$ by all walls and also by the broken line (that’s new here) to obtain toric transversality;
3. apply the degeneration formula and show that for each tropical curve there is only one possible way of gluing;
4. use the correspondence between walls and tropical disks without unbounded legs (Maslov index zero) to show that broken lines count tropical disks with one unbounded leg (Maslov index two).

Steps (1)-(3) will give the following tropical correspondence theorem:

$$\begin{aligned} N_{p,q} = p \cdot \sum _{h\in \mathfrak {H}_{p,q}(P)}Mult (h). \end{aligned}$$

Step (4) will show that coefficients of broken lines are counts of disk completions:

$$\begin{aligned} a_{\mathfrak {b}} = \sum _{h\in \mu ^{-1}(\mathfrak {b})} Mult (h). \end{aligned}$$

Together with Corollary 3.7 this gives the following formula

$$\begin{aligned} N_{p,q}=p\cdot \sum _{\mathfrak {b}\in \mathfrak {B}_{p,q}(P)}a_{\mathfrak {b}}. \end{aligned}$$

Using the definition of theta functions via broken lines this will give a proof of Theorems 1 and 2, as we will explain in detail in §5.

### Resolution of singularities

**Fig. 5:**
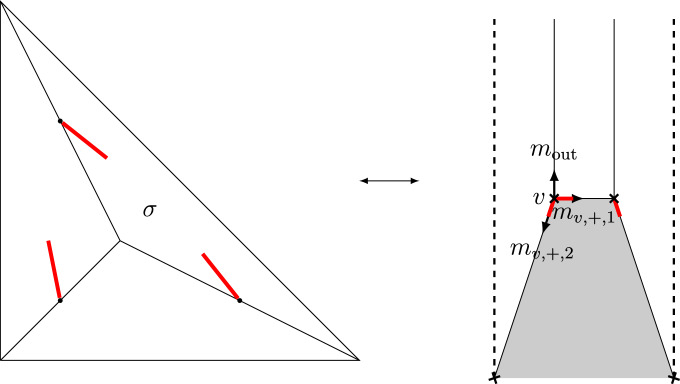
The intersection complex and its dual $(\tilde{B},\mathscr {P},\varphi )$ for a resolution of the toric degeneration of $(\mathbb {P}^2,E)$

To apply the degeneration formula we need a log smooth family. Unfortunately, the log structure of our toric degeneration is not even coherent (there is no chart for the log structure at the points corresponding to the affine singularities). However, we can obtain a log smooth degeneration without changing the general fiber by successively blowing up $\mathfrak {X}$ along the irreducible components of the central fiber. Depending on the number of components there are several ways to do this. The easiest is to choose a cyclic ordering (with respect to the intersection combinatorics) and to blow up in this order along all but one irreducible component. Figure 5 shows the resulting central fiber (intersection complex) for $\mathbb {P}^2$. The resulting family $\tilde{\mathfrak {X}}\rightarrow \mathbb {A}^1$ has general fiber *X* and is log smooth by [14], Lemma 2.12. In [8], §2, the author used a more symmetric resolution. However, this resolution is not projective, so we don’t use it here.

For later convenience we indicate the choices of small resolutions by red stubs attached to the vertices of $\mathscr {P}$. The stubs at a vertex *v* point in the directions corresponding to the toric divisors of $X_v$ intersecting an exceptional line. We denote the primitive vectors in the direction of the red stubs adjacent to *v* by $m_{v,+,i}\in \Lambda _{\tilde{B},v}$ for $i=1,\ldots ,n$, where $n\in \{0,1,2\}$ is the number of exceptional lines contained in $X_v$. Denote the primitive vectors in the direction of the other edges of $\sigma _0$ adjacent to *v* by $m_{v,-,i}\in \Lambda _{\tilde{B},v}$ for $i=1,\ldots ,n$, where $m=2-n$. Further, $m_{out }$ is the unique unbounded direction of $\tilde{B}$.

#### [Style3 Style3]Example 4.1

Figure 5 shows the intersection complex and its dual for a resolution of the toric degeneration of $(\mathbb {P}^2,E)$. One component (corresponding to $\sigma$ and *v*, respectively) contains two exceptional lines.

#### [Style1 Style1]Definition 4.2

For an effective curve class $\beta$ of *X* and $p,q\in \mathbb {N}$ with $p+q=D\cdot \beta$ define a class of stable log maps $\beta _{p,q}$ to $\tilde{\mathfrak {X}}\rightarrow \mathbb {A}^1$ as follows:

1. genus $$g=0$$;
2. fibers have curve class $\beta$;
3. 2 marked points $x_p,x_q$ having contact orders *p*, *q* with $\mathfrak {D}$.

Let $\mathscr {M}(\tilde{\mathfrak {X}}/\mathbb {A}^1,\beta _{p,q})$ be the moduli space of stable log maps to $\tilde{\mathfrak {X}}\rightarrow \mathbb {A}^1$ of class $\beta _{p,q}$. By [16] this is a proper Deligne-Mumford stack and admits a virtual fundamental class $\llbracket \mathscr {M}(\mathfrak {X}/\mathbb {A}^1,\beta _{p,q})\rrbracket$. It has virtual dimension 1, since the contact orders cut down the virtual dimension by $(p-1)+(q-1)=D\cdot \beta -2$. Let $ev : \mathscr {M}(\tilde{\mathfrak {X}}/\mathbb {A}^1,\beta _{p,q}) \rightarrow \mathfrak {D}$ be the evaluation map at $x_p$.

#### [Style1 Style1]Definition 4.3

Define the 2-marked log Gromov-Witten invariant

$$\begin{aligned} N_{p,q}(X,\beta ) = \int _{\llbracket \mathscr {M}(\mathfrak {X}/\mathbb {A}^1,\beta _{p,q})\rrbracket } ev ^\star [pt ] \in \mathbb {Q}. \end{aligned}$$

Since log Gromov-Witten invariants are constant in log smooth families ([21], Appendix A), this agrees with the definition of $N_{p,q}(X,\beta )$ in the introduction.

#### [Style3 Style3]Remark 4.4

We believe that there should be a definition of log Gromov-Witten invariants for relatively coherent targets that captures all information about the resolution described here. This would simplify the subsequent proof a lot as we wouldn’t have to translate between the toric degeneration and its resolution.

### Tropical curves and refinement

It turns out that tropical curves on $\tilde{B}$ that are tropicalizations of stable log maps to $\tilde{X}$ do not fulfill the ordinary balancing condition, but the following modified one.

#### [Style1 Style1]Definition 4.5

For $p,q\in \mathbb {Z}_{>0}$ and *P* a general point in a unbounded chamber of $\mathscr {S}_{p+q}$ let $\tilde{\mathfrak {H}}_{p,q}(P)$ be the set of tropical curves on $\tilde{B}$ with two unbounded legs, of weights *p* and *q*, with vertex of the unbounded leg of weight *p* being bivalent and mapping to *P*, and such that each vertex is of one of the following types:

(I) *V* is not mapped to a vertex of $\mathscr {P}$. Then the ordinary balancing condition holds:
  $$\begin{aligned} \sum _{E\ni V}u_{(V,E)} = 0. \end{aligned}$$
  The sum is over all edges or legs $E\in E(\Gamma _C)\cup L(\Gamma _C)$ containing *V*.
(II) *V* is mapped to a vertex *v* of $\mathscr {P}$, and is 1-valent with adjacent edge *E* mapped onto the edge of $\mathscr {P}$ containing the red stub adjacent to *v*. Then the balancing condition reads, with $m_{v,+,i}$ as in Fig. 5 for some *i*,
  $$\begin{aligned} u_{(V,E)} = km_{v,+,i}. \end{aligned}$$
(III) *V* is mapped to a vertex *v* of $\mathscr {P}$ and has exactly one adjacent edge or leg $E_{V,out }$ that is not mapped onto a compact edge of $\mathscr {P}$. All other edges (possibly none) are compact with other vertex of type (II) above. In this case, for some $k_i\ge 0$, the following balancing condition holds:
  $$\begin{aligned} \sum _{E\ni V} u_{(V,E)} + \sum _{i=1}^{n(v)}k_im_{v,+,i} = 0. \end{aligned}$$

Write $V_{(I)}(\tilde{\Gamma })$, $V_{(II)}(\tilde{\Gamma })$, $V_{(III)}(\tilde{\Gamma })$ for the set of vertices of type (I), (II), (III).

#### Lemma 4.6

**Fig. 6:**
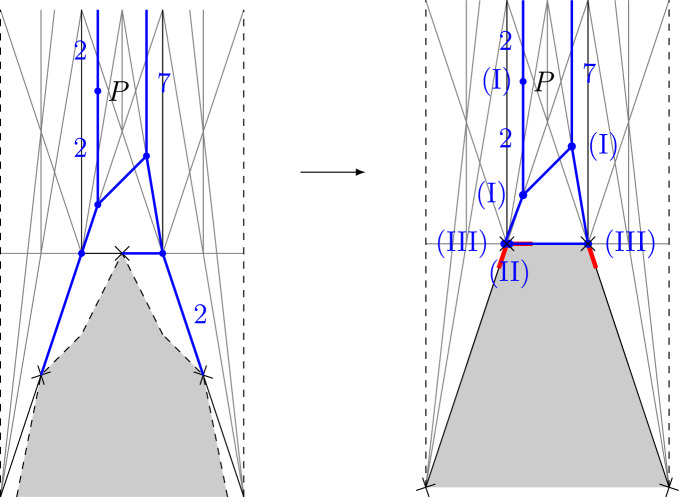
An example of the map $\mathfrak {H}_{2,7} \rightarrow \tilde{\mathfrak {H}}_{2,7}$. The weights of edges and the types (I)-(III) of vertices of the target are given. Two vertices are mapped to the same vertex, but not connected

There is a surjective map $\mathfrak {H}_{p,q}(P) \rightarrow \tilde{\mathfrak {H}}_{p,q}(P)$ by deleting bounded legs in the directions $m_{v,+,i}$ (the directions of the red stubs) and extending bounded legs in the directions $m_{v,-,i}$. See Fig. 6 for an example and [8], Construction 3.17, for details of the construction.

#### Corollary 4.7

The set $\tilde{\mathfrak {H}}_{p,q}(P)$ is finite.

#### Proof

This follows from Corollary 3.8 and Lemma 4.6. $\square$

#### [Style1 Style1]Definition 4.8

For $p,q\in \mathbb {Z}_{>0}$, a general point *P* in an unbounded chamber of $\mathscr {S}_{p+q}$ and an effective curve class $\beta$ on *X*, let $\tilde{\mathfrak {H}}_{p,q}^\beta (P)$ be the moduli space of tropical curves in $\tilde{\mathfrak {H}}_{p,q}(P)$ whose image under the map from Lemma 4.6 has class $\beta$ (see Definition 3.11. Note that

$$\begin{aligned} \tilde{\mathfrak {H}}_{p,q}(P) = \coprod _\beta \tilde{\mathfrak {H}}_{p,q}^\beta (P), \end{aligned}$$

where the sum is over all effective curve classes on *X* with $D\cdot \beta =p+q$.

#### [Style2 Style2]Proposition 4.9

$\tilde{\mathfrak {H}}_{p,q}^\beta (P)$ is the set of tropical curves that arise as tropicalizations of stable log maps in $\mathscr {M}(\tilde{X},\beta _{p,q})$. Vertices of type (II) correspond to components that are multiple covers of an exceptional $\mathbb {P}^1$, and vertices of type (III) to components intersecting an exceptional $\mathbb {P}^1$.

#### Proof

It is shown in [8], Proposition 3.12, that tropicalizations of stable log maps to $\tilde{\mathfrak {X}}$ have vertices of types (I)-(III) above. The condition to have two marked points with contact orders *p* and *q* is equivalent to the condition on the tropical curve to have two unbounded legs of weight *p* and *q*, respectively. $\square$

#### Construction 4.10

*(Refinement)* For $p,q\in \mathbb {Z}_{>0}$ let *P* be a general point in an unbounded chamber of $\mathscr {S}_{p+q}$. Let $\mathscr {P}_{p,q}$ be a refinement of $\mathscr {P}$ such that all tropical curves in $\mathfrak {H}_{p,q}(P)$ are contained in the 1-skeleton of $\mathscr {P}_{p,q}$ and *P* is a vertex of $\mathscr {P}_{p,q}$. This induces a logarithmic modification $\tilde{\mathfrak {X}}_{p,q}\rightarrow \mathbb {A}^1$ of $\tilde{\mathfrak {X}}\rightarrow \mathbb {A}^1$ via subdivision of Artin fans, see [3]. By making a base change $t\mapsto t^e$ we can scale $\mathscr {P}_{p,q}$ and thus assume it has integral vertices. By construction, all stable log maps to the central fiber *Y* of $\tilde{\mathfrak {X}}_{p,q}\rightarrow \mathbb {A}^1$ are torically transverse.

### The degeneration formula

Gromov-Witten invariants are invariant under logarithmic modifications [3]. Hence we can compute $N_{p,q}(X,\beta )$ on *Y*, the central fiber of the degeneration $\tilde{\mathfrak {X}}_{p,q}\rightarrow \mathbb {A}^1$ constructed above,

$$\begin{aligned} N_{p,q}(X,\beta ) = \int _{\llbracket \mathscr {M}(Y,\beta _{p,q})\rrbracket }ev ^\star [pt ]. \end{aligned}$$

Here $$ev $$ is the evaluation map at $$x_p$$, the marked point of order *p*.

On the central fiber *Y* we have some techniques for computing the invariants $N_{p,q}(X,\beta )$. By the decomposition formula (Proposition 4.11) the connected components of the moduli space $\mathscr {M}(Y,\beta _{p,q})$ are labelled by certain (decorated) tropical curves. The gluing formula (Proposition 4.16) relates the contributions of every tropical curve to contributions of its vertices and edges. In our case, similar to [8], the situation is particularly easy. Tropical curves in $\tilde{\mathfrak {H}}_{p,q}(P)$ have a natural orientation of edges and the gluing according to this orientation is the only one giving a nonzero contribution (Proposition 4.17).

#### [Style2 Style2]Proposition 4.11

(Decomposition formula) For $\tilde{h}\in \tilde{\mathfrak {H}}_{p,q}^\beta (P)$ let $\mathscr {M}_{\tilde{h}}$ be the moduli space of stable log maps in $\mathscr {M}(Y,\beta _{p,q})$ with tropicalization $\tilde{h}$. Then

$$\begin{aligned} \llbracket \mathscr {M}(Y,\beta _{p,q})\rrbracket = \sum _{\tilde{h}\in \tilde{\mathfrak {H}}_{p,q}^\beta (P)}\frac{l_{\tilde{\Gamma }}}{|Aut (\tilde{h})|} F_\star \llbracket \mathscr {M}_{\tilde{h}}\rrbracket , \end{aligned}$$

where $l_{\tilde{\Gamma }} := lcm \{w_E \ | \ E\in E(\tilde{\Gamma })\}$ and $F:\mathscr {M}_{\tilde{h}}\rightarrow \mathscr {M}_\beta$ is the forgetful map.

#### Proof

This is a special case of the decomposition formula of [1], similar to [8], Proposition 4.4. In [1] the sum is over tropical curves with vertices decorated by curve classes to the corresponding components. In our case, as in [8], Proposition 4.1, these curve classes are determined by the tropical curve. Hence, we can simply sum over $\tilde{\mathfrak {H}}_{p,q}(P)$. The nominator $l_{\tilde{\Gamma }}$ is the smallest integer such that scaling $\tilde{B}$ by $l_{\tilde{\Gamma }}$ leads to a tropical curve with integral vertices and edge lengths. By construction $\mathscr {P}_{p,q}$ has integral vertices and tropical curves in $\tilde{\mathfrak {H}}_{p,q}(P)$ are contained in the 1-skeleton of $\mathscr {P}_{p,q}$ with vertices mapping to vertices of $\mathscr {P}_{p,q}$. The affine length of the image of an edge *E* is a multiple of $w_E$. So the scaling necessary to obtain integral edge lengths is $l_{\tilde{\Gamma }} = lcm \{w_E \ | \ E\in E(\tilde{\Gamma })\}$. $\square$

Let $\tilde{h} : \tilde{\Gamma } \rightarrow \tilde{B}$ be a tropical curve in $\tilde{\mathfrak {H}}_{p,q}(P)$. For a vertex *V* of $\tilde{\Gamma }$ define

$$\begin{aligned} \mathscr {M}_V^\circ := \mathscr {M}(Y_{\tilde{h}(V)}^\circ ,i_V^\star \beta _{p,q}), \end{aligned}$$

where $Y_{\tilde{h}(V)}^\circ$ is the complement of the 0-dimensional toric strata in $Y_{\tilde{h}(V)}$ and $i_V : Y_{\tilde{h}(V)}^\circ \rightarrow Y$ is the inclusion map.

**Fig. 7:**
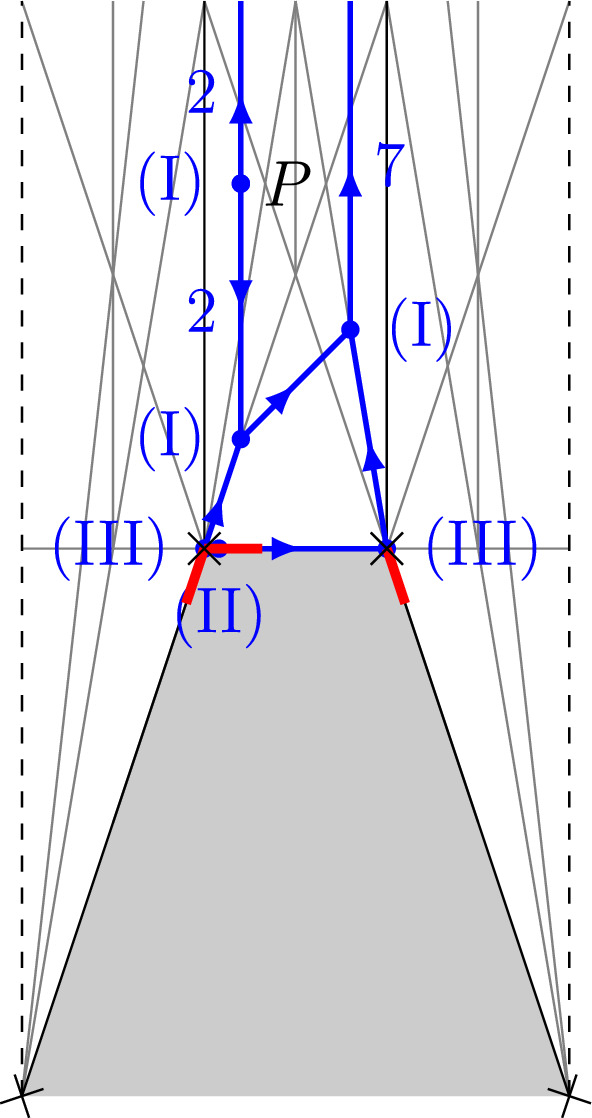
The orientation of the tropical curve from Fig. 6

For $V\in V_{II}(\tilde{\Gamma })$ (Definition 4.5) with adjacent edge *E*, the moduli space $\mathscr {M}_V^\circ$ is proper, since it is isomorphic to the moduli space of $w_E$-fold multiple covers of $\mathbb {P}^1$ totally ramified at a point. For $V\in V(\tilde{\Gamma })\setminus V_{II}(\tilde{\Gamma })$ we obtain a proper moduli space as follows. Since tropical curves in $\tilde{\mathfrak {H}}_{p,q}(P)$ have genus 0, the graph $\tilde{\Gamma }$ is a tree. We give it the structure of a rooted tree by choosing the vertex $V_{out }$ of the unbounded leg of weight *q* to be the root vertex. Then there is a natural orientation of the edges of $\tilde{\Gamma }$ by choosing edges to point from a vertex to its parent. For example, the natural orientation of the tropical curve from Fig. 6 is shown in Fig. 7. For each vertex $V\in V(\tilde{\Gamma })\setminus V_{II}(\tilde{\Gamma })$ there is an evaluation map

$$\begin{aligned} ev _{V,-}^\circ : \mathscr {M}_V^\circ \rightarrow \prod _{E\rightarrow V}D_E^\circ , \end{aligned}$$

where the product is over all edges of $\tilde{\Gamma }$ adjacent to *V* and pointing towards *V*.

#### Lemma 4.12

The evaluation map $ev _{V,-}^\circ$ is proper.

#### Proof

Combine [12], Propositions 4.2 and 5.1, as in [8], Lemma 4.5. $\square$

Since properness of morphisms is stable under base change, we obtain a proper moduli space by base change to a point $\gamma _V : Spec \mathbb {C} \rightarrow \prod _{E\rightarrow V}D_E^\circ$, that is,

$$\begin{aligned} \mathscr {M}_{\gamma _V} := Spec \mathbb {C} \times _{\prod _{E\rightarrow V}D_E^\circ } \mathscr {M}_V^\circ \end{aligned}$$

is a proper Deligne-Mumford stack.

#### Lemma 4.13

For $V\in V_{II}(\tilde{\Gamma })$ the virtual dimension of $\mathscr {M}_V$ is zero. Otherwise the virtual dimension of $\mathscr {M}_V$ equals the codimension of $\gamma _V$.

#### Proof

See [12], §5.3, and [8], Lemma 4.6. $\square$

#### [Style1 Style1]Definition 4.14

For a vertex *V* of $\tilde{\Gamma }$ define

$$\begin{aligned} N_V := \left\{ \begin{array}{ll} \int _{\llbracket \mathscr {M}_V^\circ \rrbracket }1, &  V \in V_{II}(\tilde{\Gamma }); \\ \int _{\mathscr {M}_{\gamma _V}}\gamma _V^!\llbracket \mathscr {M}_V^{\circ }\rrbracket , &  V \in V_I(\tilde{\Gamma })\cup V_{III}(\tilde{\Gamma }). \end{array}\right. \end{aligned}$$

This is a finite number by Lemma 4.13 and independent of $\gamma _V$ by Lemma 4.12.

#### [Style2 Style2]Proposition 4.15

([8], Proposition 4.8)

(I) For $V\in V_I(\tilde{\Gamma })$ let $e_1,\ldots ,e_n$ be the edges of $\mathscr {P}_{p,q}$ adjacent to $\tilde{h}(V)$ and let $m_1,\ldots ,m_n$ be the corresponding primitive vectors. Let ${\textbf {w}}_{i}=(w_{i1},\ldots ,w_{il_i})$ be the weights of edges of $\tilde{\Gamma }$ mapping to $e_i$ and write ${\textbf {w}}=({\textbf {w}}_1,\ldots ,{\textbf {w}}_n)$. Then $N_V$ is the toric invariant $N_{\textbf {m}}({\textbf {w}})$ as defined in [12] and [8], §4.1.
(II) If $V\in V_{II}(\tilde{\Gamma })$, then
  $$\begin{aligned} N_V = \frac{(-1)^{w_E-1}}{w_E^2}, \end{aligned}$$
  where *E* is the unique edge adjacent to *V*.
(III) If $V\in V_{III}(\tilde{\Gamma })$, then
  $$\begin{aligned} N_V = \sum _{i=1}^{n(h(V))}\sum _{{\textbf {w}}_{V,+}} \frac{N_{{\textbf {m}}}({\textbf {w}})}{|Aut ({\textbf {w}}_{V,+})|} \prod _{i=1}^{l_{V,i}}\frac{(-1)^{w_{V,i,j}-1}}{w_{V,i,j}}. \end{aligned}$$
  Here *n*(*h*(*V*)) is the number of red stubs attached to the vertex $v=h(V)$ of $\mathscr {P}$ (see §4.1). The second sum is over all weight vectors ${\textbf {w}}_{V,+,i}=(w_{V,i,1},\ldots ,w_{V,i,l_{V,i}})$ such that $\sum _{j=1}^{l_{V,i}} w_{V,i,j} = k_i$, with $k_i$ as in Proposition 4.9, (III). Further, $N_{{\textbf {m}}}({\textbf {w}})$ is a toric invariant as in (I) with ${\textbf {m}}=((m_{v,-,i})_i,(m_{v,+,i})_i)$ and ${\textbf {w}}=(((w_E)_{E\in E_{V,-,i})_i},({\textbf {w}}_{V,+,i})_i)$, where $E_{V,-,i}$ is the set of edges adjacent to *V* and mapped to direction $m_{v,-,i}$.

The bivalent vertex *V* mapping to *P* is of type (I) and contributes $N_V=1$.

Define  to be the moduli space of stable log maps in $\prod _V\mathscr {M}_V$ matching over the divisors $D_E$, $E\in E(\tilde{\Gamma })$, i.e., the fiber productBy [19] there is an étale morphism  of degree $deg (cut ) = (\prod _{E\in E(\tilde{\Gamma })}w_E)/l_{\tilde{\Gamma }}$, where $l_{\tilde{\Gamma }} = lcm \{w_E\}$. By compatibility of obstruction theories ([19], §9) we have

$$\begin{aligned} \llbracket \mathscr {M}_{\tilde{h}}\rrbracket = cut ^\star \delta ^!\prod _{V\in V(\tilde{\Gamma })}\llbracket \mathscr {M}_V^\circ \rrbracket . \end{aligned}$$

By the projection formula $cut _\star cut ^\star$ is multiplication with $deg (cut )$, so

$$\begin{aligned} cut _\star \llbracket \mathscr {M}_{\tilde{h}}\rrbracket = \frac{1}{\ell _{\tilde{\Gamma }}}\prod _{E\in E(\tilde{\Gamma })}w_E \cdot \delta ^!\prod _{V\in V(\tilde{\Gamma })}\llbracket \mathscr {M}_V^\circ \rrbracket . \end{aligned}$$

#### [Style2 Style2]Proposition 4.16

(Gluing formula)

$$\begin{aligned} \int _{\llbracket \mathscr {M}_{\tilde{h}}\rrbracket }1 = \frac{1}{\ell _{\tilde{\Gamma }}}\prod _{E\in E(\tilde{\Gamma })}w_E \cdot \int _{\delta ^!\prod _V\llbracket \mathscr {M}_V\rrbracket }1. \end{aligned}$$

#### Proof

By the above formula, the cycles $cut _\star \llbracket \mathscr {M}_{\tilde{h}}\rrbracket$ and $\frac{1}{\ell _{\tilde{\Gamma }}}\prod _{E\in E(\tilde{\Gamma })}w_E \cdot \delta ^!\prod _V\llbracket \mathscr {M}_V\rrbracket$ have the same restriction to the open substack . Hence their difference is rationally equivalent to a cycle supported on the closed substack . Suppose there exists an element $(f_V : C_V \rightarrow Y_{\tilde{h}(V)})_{V\in V(\tilde{\Gamma })}\in Z$. Then at least one of the source curves $C_V$ would contain a nontrivial cycle of components as can be seen by a loop construction as in the proof of [12], Proposition 4.2, or [8], Lemma 4.5. This contradicts $g=0$, so *Z* is empty, completing the proof. $\square$

#### [Style2 Style2]Proposition 4.17

(Unique gluing)

$$\begin{aligned} \int _{\delta ^!\prod _{V}\llbracket \mathscr {M}_V\rrbracket } 1 = \prod _{V\in V(\tilde{\Gamma })}N_V. \end{aligned}$$

#### Proof

This is similar to the proof of [8], Proposition 4.13. As before, $\tilde{\Gamma }$ is a rooted tree with root vertex $V_{out }$. This gives an orientation of the edges of $\tilde{\Gamma }$. The only gluing that gives a nonzero contribution after integration is the one according to this orientation. Any other gluing gives a negative virtual dimension, since we have too many conditions on one of the irreducible components. $\square$

#### [Style2 Style2]Proposition 4.18

(Degeneration formula) Let *P* be a point in an unbounded chamber of $\mathscr {S}_{p+q}$. Then

$$\begin{aligned} N_{p,q}(X,\beta ) = \sum _{\tilde{h}\in \tilde{\mathfrak {H}}_{p,q}^\beta (P)} \frac{1}{|Aut (\tilde{h})|} \cdot \prod _{E\in E(\tilde{\Gamma })}w_E \cdot \prod _{V\in V(\tilde{\Gamma })} N_V. \end{aligned}$$

#### Proof

Since the virtual dimension of $\mathscr {M}_\beta$ is zero, integration (i.e., proper pushforward to a point) of the decomposition formula (Proposition 4.11) gives

$$\begin{aligned} N_{p,q}(X,\beta ) = \sum _{\tilde{h}\in \tilde{\mathfrak {H}}_{p,q}^\beta (P)} \frac{1}{|Aut (\tilde{h})|}\int _{\llbracket \mathscr {M}_{\tilde{h}}\rrbracket } 1. \end{aligned}$$

Using Propositions 4.16 and 4.17 we get the above formula. $\square$

We get a more symmetric formula by summing over balanced tropical curves:

#### [Style1 Style1]Definition 4.19

Let $h : \Gamma \rightarrow B$ be a tropical curve in $\mathfrak {H}_\beta$ and let *V* be a vertex of $\Gamma$. Then the image of *V* under the map from Lemma 4.6 is a vertex of $\tilde{\Gamma }$ of type (I) or (III). Let ${\textbf {m}}$ and ${\textbf {w}}$ be as in the respective case of Proposition 4.15 and define $N_V^{tor } := N_{{\textbf {m}}}({\textbf {w}}).$ Note that $N_V^{tor }=N_V$ for vertices of type (I).

#### [Style1 Style1]Definition 4.20

Define $N_{p,q}^{trop }(X,\beta )=\sum _{h\in \mathfrak {H}_{p,q}^\beta (P)} N_h$, where *P* is a point in an unbounded chamber of $\mathscr {S}_{p+q}$ and

$$\begin{aligned} N_h := \frac{1}{|Aut (h)|} \cdot \prod _{E\in E(\Gamma )}w_E\cdot \prod _{E\in L_\Delta (\Gamma )}\frac{(-1)^{w_E-1}}{w_E}\cdot \prod _{V\in V(\Gamma )} N_V^{tor }. \end{aligned}$$

Here $L_\Delta (\Gamma )$ is the set of bounded legs of $\Gamma$.

Recall the definition of tropical mutliplicity from Definition 3.9.

#### [Style2 Style2]Proposition 4.21

For a tropical curve $h : \Gamma \rightarrow B$ in $\mathfrak {H}_q$ we have

$$\begin{aligned} N_h = Mult (h) \end{aligned}$$

#### Proof

By the tropical correspondence theorem with point conditions on toric divisors ([12], Theorem 3.4) we have $m_V = \prod _{E\rightarrow V}w_E \cdot N_V^{tor }$. The product is over all eges of $\Gamma$ pointing towards *V* with respect to the orientation of $\Gamma$ such that all edges point towards the root vertex $V_{out }$. Then

$$\begin{aligned} \prod _{V\in V(\Gamma )}m_V = \prod _{E\in E(\Gamma )} w_E \cdot \prod _{V\in V(\Gamma )} N_V^{tor }, \end{aligned}$$

as each $E\in E(\Gamma )$ occurs exactly once. Plugging this and $m_E=(-1)^{w_E+1}/w_E^2$ into the definition of $Mult (h)$ we obtain $N_h$. $\square$

#### Theorem 4.22

(Tropical correspondence theorem)

$$\begin{aligned} N_{p,q}^{trop }(X,\beta ) = pN_{p,q}(X,\beta ). \end{aligned}$$

#### Proof

This follows from a rearrangement of equations similar to the proof of [8], Theorem 4.17:

Using Propositions 4.18 and 4.15, we have

$$\begin{aligned} N_{p,q}(X,\beta )= &   \sum _{\tilde{h}\in \tilde{\mathfrak {H}}_\beta }\left( \frac{1}{|Aut (\tilde{h})|} \cdot \prod _{E\in E(\tilde{\Gamma })} w_E \cdot \prod _{V\in V_I(\tilde{\Gamma })}N_V^{tor } \cdot \prod _{V\in V_{II}(\tilde{\Gamma })}\frac{(-1)^{w_{E_V}-1}}{w_{E_V}^2}\right. \\  &   \hspace{0cm} \left. \cdot \prod _{V\in V_{III}(\tilde{\Gamma })}\left( \sum _{i=1}^{n(v)}\sum _{{\textbf {w}}_{V,+}} N_V^{tor } \frac{1}{|Aut ({\textbf {w}}_{V,+})|}\prod _{i=1}^{n(h(V))}\prod _{j=1}^{l_{V,i}}\frac{(-1)^{w_{V,i,j}-1}}{w_{V,i,j}}\right) \right) . \end{aligned}$$

Canceling the $w_{E_V}$ for vertices of type (II) in the first product against the ones in the denominator of the third product and factoring out the second sum we get

$$\begin{aligned} N_{p,q}(X,\beta )= &   \sum _{\tilde{h}\in \tilde{\mathfrak {H}}_{p,q}^\beta (P)}\sum _{({\textbf {w}}_{V,+,i})_{V,i}} \left( \frac{1}{|Aut (\tilde{h})||Aut ({\textbf {w}}_{V,+})|} \cdot \prod _{E\in E(\tilde{\Gamma })\setminus \cup _{V\in V_{II}(\tilde{\Gamma })}\{E_V\}}w_E \right. \\    &   \hspace{-2cm} \left. \cdot \prod _{V\in V_I(\tilde{\Gamma })\cup V_{III}(\tilde{\Gamma })} N_V^{tor } \cdot \prod _{V\in V_{II}(\tilde{\Gamma })}\frac{(-1)^{w_{E_V}-1}}{w_{E_V}}\cdot \prod _{V\in V_{III}(\tilde{\Gamma })}\prod _{i=1}^{n(h(V))}\prod _{j=1}^{l_{V,i}}\frac{(-1)^{w_{V,i,j}-1}}{w_{V,i,j}}\right) . \end{aligned}$$

The second sum is over all collections $({\textbf {w}}_{V,+,i})_{i,V}$ indexed by $V\in V_{III}(\tilde{\Gamma })$ and $i=1,\ldots ,n(h(V))$ of weight vectors ${\textbf {w}}_{V,+,i}=(w_{V,i,1},\ldots ,w_{V,i,l_{V,i}})$ such that $\sum _{j=1}^{l_{V,i}} w_{V,i,j} = k_i$, with $k_i$ as in Proposition 4.9, (III). By the construction of the map $\mathfrak {H}_d \rightarrow \tilde{\mathfrak {H}}_d$ in Lemma 4.6, the two summations can be replaced by a summation over $\mathfrak {H}_d$. Note that for $\tilde{h}\in \tilde{\mathfrak {H}}_d$ we have

$$\begin{aligned} \sum _{h\mapsto \tilde{h}}\frac{1}{|Aut (h)|} = \frac{1}{|Aut (\tilde{h})|}\sum _{({\textbf {w}}_{V,+})_{V\in V_{III}(\tilde{\Gamma })}} \frac{1}{|Aut ({\textbf {w}}_{V,+})|}, \end{aligned}$$

where the sum is over all $h\in \mathfrak {H}_d$ giving $\tilde{h}$ via the map from Lemma 4.6. This can be seen by multiplying both sides with $|\text {Aut}(\tilde{h})|$. Moreover, note that $V(\Gamma )=V_I(\tilde{\Gamma })\cup V_{III}(\tilde{\Gamma })$ and $E(\Gamma )=E(\tilde{\Gamma })\setminus \cup _{V\in V_{II}(\tilde{\Gamma })}\{E_V\}$, where, for a vertex *V* of type (II), $E_V$ is the unique edge containing the vertex *V*. Then

$$\begin{aligned} N_{p,q}(X,\beta )= &   \sum _{h\in \mathfrak {H}_\beta }\left( \frac{1}{|Aut (h)|} \cdot \prod _{E\in E(\Gamma )} w_E \cdot \prod _{V\in V(\Gamma )} N_V^{tor } \right. \\    &   \hspace{1cm} \left. \cdot \prod _{V\in V_{II}(\tilde{\Gamma })}\frac{(-1)^{w_{E_V}-1}}{w_{E_V}}\cdot \prod _{V\in V_{III}(\tilde{\Gamma })}\prod _{i=1}^{n(h(V))}\prod _{j=1}^{l_{V,i}}\frac{(-1)^{w_{V,i,j}-1}}{w_{V,i,j}} \right) . \end{aligned}$$

Using that

$$\begin{aligned} \prod _{E\in L_\Delta (\Gamma )} \frac{(-1)^{w_E-1}}{w_E} = \prod _{V\in V_{II}(\tilde{\Gamma })}\frac{(-1)^{w_{E_V}-1}}{w_{E_V}}\cdot \prod _{V\in V_{III}(\tilde{\Gamma })}\prod _{i=1}^{n(h(V))}\prod _{j=1}^{l_{V,i}}\frac{(-1)^{w_{V,i,j}-1}}{w_{V,i,j}} \end{aligned}$$

completes the proof. $\square$

### Broken line calculations

#### [Style1 Style1]Definition 4.23

Let $\mathfrak {p}\in \mathscr {S}_\infty$ be a wall and choose $x\in Int (\mathfrak {p})$. Define $\mathfrak {H}_{\mathfrak {p},w}^\circ$ to be the set of all tropical disks $h^\circ : \Gamma \rightarrow B$ with $h^\circ (V_\infty )=x$ and $u_{(V_\infty ,E_\infty )}=-w\cdot m_{\mathfrak {p}}$. Note that the sets $\mathfrak {H}_{\mathfrak {p},w}$ are in bijection for different choices of $x\in Int (\mathfrak {p})$. For $h^\circ \in \mathfrak {H}_{\mathfrak {p},w}^\circ$ define $Mult (h^\circ )$ as in Definition 4.20.

#### Lemma 4.24

([8], Proposition 5.20) For a wall $\mathfrak {p}$ of $\mathscr {S}_\infty$ we have

$$\begin{aligned} log f_{\mathfrak {p}} = \sum _{w=1}^\infty \sum _{h^\circ \in \mathfrak {H}_{\mathfrak {p},w}^\circ } wMult (h^\circ ) z^{(wm_{\mathfrak {p}},0)}. \end{aligned}$$

#### [Style2 Style2]Proposition 4.25

([6], Proposition 6.15)

$$\begin{aligned} a_{\mathfrak {b}} = \sum _{h\in \mu ^{-1}(\mathfrak {b})} Mult (h). \end{aligned}$$

#### Proof

Consider a break point $P'$ of $\mathfrak {b}$ and let $az^m, a'z^{m'}$ be the monomials attached to the line segments before and after the breaking, respectively. To complete $\mathfrak {b}$ (Proposition 3.2) at $P'$ we have to add one or more tropical disks ending in $P'$ such that the sum of their ending weights $u_{(V_\infty ,E_\infty )}$ equals $\bar{m}+\bar{m}'$. Let $\mathfrak {H}_{\mathfrak {b}}(P')$ be the set of such collections and for $(h_1^\circ ,\ldots ,h_s^\circ )\in \mathfrak {H}_{\mathfrak {b}}(P')$ let $m_{V_\infty }$ be the multiplicity of the vertex mapping to $P'$ for any completion $h\in \mu ^{-1}(\mathfrak {b})$ involving $(h_1^\circ ,\ldots ,h_s^\circ )$. This is well-defined, since $m_{V_\infty }$ depends only on $(h_1^\circ ,\ldots ,h_s^\circ )$ and not on tropical disks we add to the other break points of $\mathfrak {b}$.

Claim:

$$\begin{aligned} \frac{a'}{a} = \sum _{(h_1^\circ ,\ldots ,h_s^\circ )\in \mathfrak {H}_{\mathfrak {b}}(P')} m_{V_\infty }\prod _{i=1}^s \text {Mult}(h_i^\circ ). \end{aligned}$$

Let $\mathfrak {p}$ be the wall containing the break point $P'$ and define $a_{\mathfrak {p}}=|\bar{m}\wedge \bar{m}'|\text {log }f_{\mathfrak {p}}$. By definition $\frac{a'}{a}$ is the coefficient of $z^{m-m'}$ in $\text {exp}(a_{\mathfrak {p}})$. By Lemma 4.24, $a_{\mathfrak {p}}$ is the generating function of $|\bar{m}\wedge \bar{m}'|w_{E_\infty }Mult (h^\circ )$ for tropical disks ending in $P'$. Hence, by standard combinatorial arguments, $\text {exp}(a_{\mathfrak {p}})$ is a sum over collections of such tropical disks. Hence, the coefficient of $z^{m-m'}$ is given by

$$\begin{aligned} \frac{a'}{a} = \sum _{(h_1^\circ ,\ldots ,h_s^\circ )\in \mathfrak {H}_{\mathfrak {b}}(P')}\prod _{i=1}^s |\bar{m}\wedge \bar{m}'|w_{E_\infty }(h_s^\circ ) \cdot Mult (h_s^\circ ). \end{aligned}$$

But $|\bar{m}\wedge \bar{m}'|\prod _{i=1}^s w_{E_\infty }(h_s^\circ )$ is nothing but $m_{V_\infty }$. This proves the claim.

Now let $a_1z^{m_1},\ldots ,a_rz^{m_r}$ be all the monomials attached to line segments of $\mathfrak {b}$ and for $i=1,\ldots ,r-1$ let $P_i$ be the break point between $a_iz^{m_i}$ and $a_{i+1}z^{m_{i+1}}$. We can expand

$$\begin{aligned} a_{\mathfrak {b}} := a_r = \frac{a_r}{a_{r-1}}\frac{a_{r-1}}{a_{r-2}}\cdots \frac{a_2}{a_1}a_1. \end{aligned}$$

By definition $a_1=1$ and for each fraction we can use the claim to obtain

$$\begin{aligned} a_{\mathfrak {b}} = \prod _{i=1}^{r-1} \sum _{(h_1^\circ ,\ldots ,h_s^\circ )\in \mathfrak {H}_{\mathfrak {b}}(P_i)} m_{V_\infty }\prod _{i=1}^s\text {Mult}(h_i^\circ ). \end{aligned}$$

The first product and summation can be replaced by a summation over all possible combinations of collections $(h_1^\circ ,\ldots ,h_s^\circ )$ for all $P_i$. But this is nothing but a choice of completion $h\in \mu ^{-1}(\mathfrak {b})$. Moreover, the product of the $m_{V_\infty }\prod _{i=1}^s\text {Mult}(h_i^\circ )$ is nothing but $\text {Mult}(h)$. Hence, we get the formula claimed in the proposition. $\square$

## Theta functions

### [Style1 Style1]Definition 5.1

For a point $P\in B_0$ and an $q\in \mathbb {N}$ define the corresponding *theta function* by

$$\begin{aligned} \vartheta _q(P) = \sum _{\mathfrak {b}\in \mathfrak {B}_q(P)} a_{\mathfrak {b}} z^{m_{\mathfrak {b}}} \end{aligned}$$

### [Style2 Style2]Proposition 5.2

([11], Theorem 3.24, [17], Theorem 1.9) Theta functions generate a commutative ring (associative if *X* is Fano) with unit $\vartheta _0$ by the multiplication rule

$$\begin{aligned} \vartheta _p(P) \cdot \vartheta _q(P) = \sum _{r=0}^\infty \alpha _{p,q}^r(P) \vartheta _r(P) \end{aligned}$$

with structure constants

$$\begin{aligned} \alpha _{p,q}^r(P) = \sum _{\begin{array}{c} (\mathfrak {b}_1,\mathfrak {b}_2)\in \mathfrak {B}_p(P)\times \mathfrak {B}_q(P) \\ m_{\mathfrak {b}_1}+m_{\mathfrak {b}_2}= \ r \end{array}} a_{\mathfrak {b}_1}a_{\mathfrak {b}_2} \end{aligned}$$

### Theorem 5.3

(Theorems 1 and 2) Write $x=z^{(-m_{out },-1)}$ and $t=z^{(0,1)}$. Then

1 $$\begin{aligned} \vartheta _q = x^{-q} + \sum _{p=1}^\infty pN_{p,q}t^{p+q}x^p \end{aligned}$$

Moreover, $\alpha _{p,q}^{r}=1$ if $r=p+q$ and otherwise

2 $$\begin{aligned} \alpha _{p,q}^r = ((p-r)N_{p-r,q} + (q-r)N_{q-r,p})t^{p+q-r}, \end{aligned}$$

where we define $N_{p,q}=0$ whenever $p\le 0$.

### Proof

This follows from Theorem 4.22 and Proposition 4.25. $\square$

Plugging both expressions of Theorem 5.3 into the multiplication rule we obtain relations among the $N_{p,q}$. Since powers of $\vartheta _1$ generate the theta ring these equations determine all $N_{p,q}$ by only knowing the invariants $N_{p,1}$ or, equivalently, the invariants $N_{1,q}$.

### [Style3 Style3]Example 5.4

We use Theorem 5.3 to obtain relations among the 2-marked invariants $N_{p,q}$ for $\mathbb {P}^2$ up to order $d=(p+q)/3=2$. By (3) we have

$$\begin{aligned} \vartheta _1= &   x^{-1}+2N_{2,1}t^3x^2+5N_{5,1}t^6x^5+\mathcal {O}(t^9) \\ \vartheta _2= &   x^{-2}+N_{1,2}t^3x+4N_{4,2}t^6x^4+\mathcal {O}(t^9) \\ \vartheta _3= &   x^{-3}+3N_{3,3}t^6x^3+\mathcal {O}(t^9) \\ \vartheta _4= &   x^{-4}+2N_{2,4}t^6x^2+\mathcal {O}(t^9) \\ \vartheta _5= &   x^{-5}+N_{1,5}t^6x+\mathcal {O}(t^9) \end{aligned}$$

By direct mutliplication we get

$$\begin{aligned} \vartheta _1\cdot \vartheta _1=x^{-2}+4N_{2,1}t^3x+(4N_{2,1}^2+10N_{5,1})t^6x^4+\mathcal {O}(t^9). \end{aligned}$$

On the other hand, (4) gives, with $\alpha _{1,1}^1=0$,

$$\begin{aligned} \vartheta _1\cdot \vartheta _1 = \vartheta _2 = x^{-2}+N_{1,2}t^3x+4N_{4,2}t^6x^4 + \mathcal {O}(t^9) \end{aligned}$$

Comparing these two equations we get the relations

$$\begin{aligned} N_{1,2}=4N_{2,1}, \qquad 2N_{4,2}=2N_{2,1}^2+5N_{5,1} \end{aligned}$$

Similarly, comparing

$$\begin{aligned} \vartheta _1\cdot \vartheta _2=x^{-3}+(N_{1,2}+2N_{2,1})t^3x^0+(2N_{1,2}N_{2,1}+4N_{4,2}+5N_{5,1})t^6x^3+\mathcal {O}(t^9) \end{aligned}$$

with

$$\begin{aligned} \vartheta _1\cdot \vartheta _2=\vartheta _3+(N_{1,2}+2N_{2,1})t^3\vartheta _0=x^{-3}+(N_{1,2}+2N_{2,1})x^0+3N_{3,3}x^3+\mathcal {O}(t^9) \end{aligned}$$

we get

$$\begin{aligned} 3N_{3,3} = 2N_{1,2}N_{2,1}+4N_{4,2}+5N_{5,1}. \end{aligned}$$

Comparing

$$\begin{aligned} \vartheta _1\cdot \vartheta _3=x^{-4}+2N_{2,1}t^3x^{-1}+(3N_{3,3}+5N_{5,1})t^6x^2+\mathcal {O}(t^9) \end{aligned}$$

with

$$\begin{aligned} \vartheta _1\cdot \vartheta _3=\vartheta _4+2N_{2,1}t^3\vartheta _1=x^{-4}+2N_{2,1}t^3x^{-1} +(4N_{2,1}^2+2N_{2,4})t^6x^2+\mathcal {O}(t^9) \end{aligned}$$

we get the relation

$$\begin{aligned} 3N_{3,3}+5N_{5,1}=4N_{2,1}^2+2N_{2,4} \end{aligned}$$

and comparing

$$\begin{aligned} \vartheta _1\cdot \vartheta _4 = x^{-5}+2N_{2,1}t^3x^{-2}+(2N_{2,4}+5N_{5,1})t^6x+\mathcal {O}(t^9) \end{aligned}$$

with

$$\begin{aligned} \vartheta _1\cdot \vartheta _4 = \vartheta _5+2N_{2,1}t^3\vartheta _2 = x^{-5}+2N_{2,1}t^3x^{-2}+(N_{1,5}+2N_{1,2}N_{2,1})t^6x+\mathcal {O}(t^9) \end{aligned}$$

we get

$$\begin{aligned} 2N_{2,4}+5N_{5,1}=N_{1,5}+2N_{1,2}N_{2,1}. \end{aligned}$$

Knowing $N_{1,2}=4$ and $N_{1,5}=25$, e.g. by direct computation as in §7, we can solve the above equations and get $N_{2,1}=1$ as well as

$$\begin{aligned} N_{2,4}=14,\qquad N_{3,3}=9,\qquad N_{4,2}=\frac{7}{2},\qquad N_{5,1}=1. \end{aligned}$$

## Higher genus and $\varvec{q}$-refined invariants

For an effective curve class $\underline{\beta }$ of *X* let $\beta _{p,q}^g$ be the class of stable log maps to *X* of genus *g*, class $\underline{\beta }$ and two marked points with contact orders *p* and *q* with *D*. The moduli space $\mathscr {M}(X,\beta _{p,q}^g)$ of basic stable log maps of class $\beta _{p,q}^g$ has virtual dimension $g+1$. We cut this dimension down to zero by fixing the image of the first marked point and inserting a *lambda class*. Let $\pi : \mathcal {C} \rightarrow \mathscr {M}(X,\beta _{p,q}^g)$ be the universal curve, of relative dualizing sheaf $\omega _\pi$. Then $\mathbb {E}=\pi _\star \omega _\pi$ is a rank *g* vector bundle over $\mathscr {M}(X,\beta ^g)$, called the Hodge bundle. The lambda classes are the Chern classes of the Hodge bundle, $\lambda _j=c_j(\mathbb {E})$. Let $ev : \mathscr {M}(X,\beta _{p,q}^g) \rightarrow D$ be the evaluation map at the marked point of order *p*. Define the 2-marked log Gromov-Witten invariant

$$\begin{aligned} N_{p,q}^g(X,\beta ) = \int _{\llbracket \mathscr {M}(X,\beta _{p,q}^g)\rrbracket } (-1)^g\lambda _g ev ^\star [pt ] \in \mathbb {Q}. \end{aligned}$$

### [Style1 Style1]Definition 6.1

Let $h : \Gamma \rightarrow B$ be a tropical curve. For a trivalent vertex *V* with multiplicity $m_V$ (Definition 3.9) define, with $\varvec{q}=e^{i\hbar }$,

$$\begin{aligned} m_V(\varvec{q}) = \frac{1}{i\hbar }\left( \varvec{q}^{m_V/2}-\varvec{q}^{-m_V/2}\right) . \end{aligned}$$

For a vertex with higher valvency define $m_V(\varvec{q}) = \prod _{V'\in V(h'_V)} m_{V'}(\varvec{q})$ with $h'_V$ as in Definition 3.9. For a bounded leg *E* with weight $w_E$ define

$$\begin{aligned} m_E(\varvec{q}) = \frac{(-1)^{w_E}}{w_E}\cdot \frac{i\hbar }{\varvec{q}^{w_E/2}-\varvec{q}^{-w_E/2}}. \end{aligned}$$

Then define the $\varvec{q}$-*refined multiplicity* of *h* to be

$$\begin{aligned} m_h(\varvec{q}) = \frac{1}{|Aut (h)|} \cdot \prod _{V\in V(\Gamma )} m_V(\varvec{q}) \cdot \prod _{E\in L_\Delta (\Gamma )} m_E(\varvec{q}). \end{aligned}$$

### Theorem 6.2

Let *P* be a point in an unbounded chamber of $\mathscr {S}_{p+q}$. Then, with $\varvec{q}=e^{i\hbar }$,

$$\begin{aligned} \sum _{g\ge 0} N_{p,q}^g(X,\beta )\hbar ^{2g} = \sum _{h\in \mathfrak {H}_{p,q}^\beta (P)} m_h(\varvec{q}) \end{aligned}$$

### Proof

Consider a stable log map in $\mathscr {M}(X,\beta ^g)$ and let $h : \Gamma \rightarrow B$ be its tropicalization. The genus of *h* is $g_h = g_\Gamma + \sum _V g_V$, where $g_\Gamma$ is the genus of the graph $\Gamma$ and $g_V$ is the genus attached to a vertex *V*. Using gluing and vanishing properties of lambda classes, Bousseau showed in [4] that $\Gamma$ is still a tree ($g_\Gamma =0$), i.e., all contributions to $g_h$ come from vertices. Hence, *h* maps to an element of $\mathfrak {H}_\beta$ by forgetting genera at vertices $g_V$. So we can sum over $\mathfrak {H}_{p,q}^\beta (P)$, but have to consider $\varvec{q}$-refined contributions of vertices. By [4], Proposition 29, the contribution of a vertex *V* with classical multiplicity $m_V$ is $m_V(\varvec{q})$. By [5], Proposition 5.1, the contribution of a bounded leg *L* is $m_L(\varvec{q})$. $\square$

To obtain a higher genus version of Theorem 1, we have to $\varvec{q}$-refine the slab functions in the initial wall structure $\mathscr {S}_0$. For $\varvec{q}$-refined wall structures it turns out to be more convenient to work with the logarithm of such functions. Define the $\varvec{q}$-refined initial wall structure $\mathscr {S}_0(\varvec{q})$ to have the same slabs as $\mathscr {S}_0$ but with slab functions $f_{\mathfrak {p}}=1+z^{(m,0)}$ replaced by $f_{\mathfrak {p}}(\varvec{q})$, where

$$\begin{aligned} log f_{\mathfrak {p}}(\varvec{q}) = \sum _{k\ge 1}\frac{(-1)^{k+1}i\hbar }{\varvec{q}^{k/2}-\varvec{q}^{-k/2}}z^{(km,0)}. \end{aligned}$$

The coefficient of $z^{(km,0)}$ is the $\varvec{q}$-multiplicity of a bounded leg of weight *k*.

The inductive construction of wall structures and broken lines can be performed with ${\textbf {q}}$-refined objects. The formulas are the same, the only difference being that all functions carry an additional $\hbar$-variable. Hence, for each finite order $k>0$ we obtain a ${\textbf {q}}$-refined wall structure $\mathscr {S}_k(\varvec{q})$. Let $\mathscr {S}_\infty (\varvec{q})$ denote the limit $k\rightarrow \infty$.

**Fig. 8:**
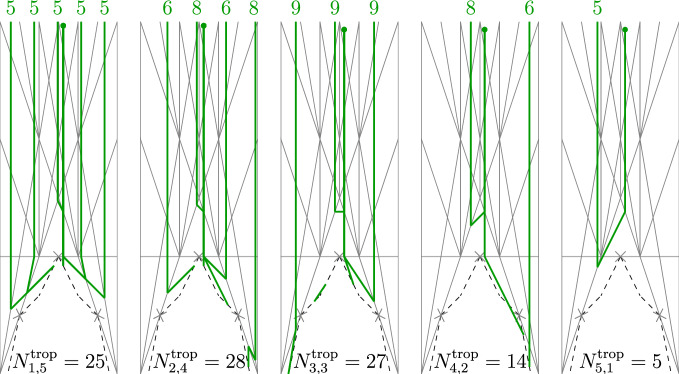
Broken lines used to compute $N_{p,q}(\mathbb {P}^2,2L)$. The numbers above the infinite segments are the coefficients $a_{\mathfrak {b}}$

Let *P* be a point in an unbounded chamber of $\mathscr {S}_{p+q}(\varvec{q})$. Then a broken line $\mathfrak {b}\in \mathfrak {B}_{p,q}(P)$ has ending monomial $a_{\mathfrak {b}}(\varvec{q})t^{p+q}x^p$ for $x=z^{(-m_{\text {out}},-1)}$. Define $\vartheta _q(\varvec{q})$ and $\alpha _{p,q}^r(\varvec{q})$ similar to $\vartheta _q$ and $\alpha _{p,q}^r$ in §5, but with $\varvec{q}$-refined broken lines.

### Theorem 6.3

(Higher genus version of Theorems 1 and 2) Write $x=z^{(-m_{out },-1)}$ and $t=x^{(0,1)}$. Then

3 $$\begin{aligned} \vartheta _q(\varvec{q}) = x^{-q} + \sum _{p=1}^\infty \sum _{g\ge 0} pN_{p,q}^g \hbar ^{2g}t^{p+q}x^p \end{aligned}$$

Moreover, $\alpha _{p,q}^{r}(\varvec{q})=1$ if $r=p+q$ and otherwise

4 $$\begin{aligned} \alpha _{p,q}^r(\varvec{q}) = \sum _{g\ge 0}((p-r)N_{p-r,q}^g + (q-r)N_{q-r,p}^g)\hbar ^{2g}t^{p+q-r}, \end{aligned}$$

where we define $N_{p,q}^g=0$ whenever $p\le 0$.

### Proof

By [8], we have $log f_{\mathfrak {p}}(\varvec{q}) = \sum _{w=1}^\infty \sum _{h\in \mathfrak {H}_{\mathfrak {p},w}} m_h(\varvec{q}) z^{(wm_{\mathfrak {p}},0)}$, the $\varvec{q}$-refined version of Lemma 4.24. As a consequence, we get a $\varvec{q}$-refined version of Proposition 4.25: $a_{\mathfrak {b}}(\varvec{q}) = \sum _{h\in \mu ^{-1}(\mathfrak {b})} m_h(\varvec{q})$. Now the statement follows from the definitions of $\vartheta _q(\varvec{q})$ and $\alpha _{p,q}^r(\varvec{q})$. $\square$

## Example calculations

We use a sage code to calculate the numbers $N_{1,3d-1}(\mathbb {P}^2,dL)$ for $d\le 4$. It can be found on timgraefnitz.com. In the code, broken lines are implemented in reversed order. We start with point *P* on an unbounded maximal cell of $\mathscr {S}_{3d}(\mathbb {P}^2)$ and a broken line coming out of this point in the negative of the unique unbounded direction $m_{out }$, with attached monomial $z^{qm_{out }}$. We can do this, since all broken lines that end in *P* have to be parallel to $m_{out }$. When the broken line hits a wall, we apply the transformation $z^m \mapsto f^{{\left\langle {n,\bar{m}}\right\rangle }}z^m$, where *n* is the normal direction of the wall. Each term in $f^{{\left\langle {n,\bar{m}}\right\rangle }}z^m$ gives a new possible broken line. The above procedure is applied recursively until the direction of the new broken line is $m_{out }$. Then we have found a broken line with asymptotic monomial $z^{qm_{out }}$ and ending in *P*. If we add together the coefficients $a_{\mathfrak {b}}$ of the broken lines $\mathfrak {b}$ with asymptotic monomial $z^{qm_{out }}$ and resulting monomial $a_{\mathfrak {b}}z^{-pm_{out }}$ we get the tropical count $N_{p,q}^{trop }(\mathbb {P}^2,dL)$, where $d=(p+q)/3$. Using the tropical correspondence (Theorem 4.22) we obtain the 2-marked log Gromov-Witten invariants $N_{p,q}=N_{p,q}(\mathbb {P}^2,dL)$. Figure 8 shows the broken lines for $d=2$. This gives

**Table:** 

$N_{1,5}=25$	$N_{2,4}=14$	$N_{3,3}=9$	$N_{4,2}=\frac{7}{2}$	$N_{5,1}=1$

## Footnotes

1 The slope of a wall is ${\left\langle {m_{\mathfrak {p}},m_{\text {out}}}\right\rangle }$, where $m_{\mathfrak {p}}$ is the primitive integral tangent vector of the wall $\mathfrak {p}$ and the scalar product ${\left\langle {-,-}\right\rangle }$ is induced from the affine structure which locally gives an affine linear emebdding into $\mathbb {R}^2$.

## Declarations

### Data availability

The sage code used for the calculations is openly available at the author’s webpage https://timgraefnitz.com/.
